# O Papel dos Níveis Séricos de ANP na Perda de Peso, Risco Cardiometabólico e Composição Corporal de Adolescentes com Obesidade Submetidos a Terapia Interdisciplinar

**DOI:** 10.36660/abc.20200735

**Published:** 2021-11-17

**Authors:** Ana Claudia Pelissari Kravchychyn, Raquel Munhoz da Silveira Campos, Yasmin Alaby Martins Ferreira, Sofia Emanuelle de Castro Ferreira Vicente, Flávia Campos Corgosinho, Lila Missae Oyama, Valter Tadeu Boldarine, Lian Tock, David Thivel, Ana Raimunda Dâmaso

**Affiliations:** 1 Universidade Federal de São Paulo Programa de Pós-graduação em Nutrição São Paulo SP Brasil Universidade Federal de São Paulo - Programa de Pós-graduação em Nutrição , São Paulo , SP – Brasil; 2 Universidade Federal de São Paulo Departamento de Biociências Programa de Pós Graduação Interdisciplinar em Ciências da Saúde Santos SP Brasil Universidade Federal de São Paulo - Departamento de Biociências Campus Baixada Santista - Programa de Pós Graduação Interdisciplinar em Ciências da Saúde , Santos , SP – Brasil; 3 Anhanguera Educacional Ltda Guarulhos SP Brasil Anhanguera Educacional Ltda , Guarulhos , SP – Brasil; 4 Universidade Federal de Goiás Goiânia GO Brasil Universidade Federal de Goiás – Nutrição, Goiânia , GO – Brasil; 5 Université Clermont Auvergne Clermont-Ferrand Auvergne-Rhône-Alpes França Université Clermont Auvergne , Clermont-Ferrand , Auvergne-Rhône-Alpes – França

**Keywords:** Síndrome Metabólica, Peptídeo Natriurético Atrial, Obesidade, Adolescente, Perda de Peso, Resistência à Insulina, Metabolismo

## Abstract

**Fundamento:**

A ação do peptídeo natriurético atrial (ANP) na natriurese, diurese e vasodilatação, resistência à insulina, fígado, rim e tecido adiposo pode contribuir para o desenvolvimento metabólico e cardiovascular saudável. Embora o nível circulante de ANP seja reduzido em pacientes com obesidade, sua resposta à perda de peso ainda é pouco explorada em populações pediátricas.

**Objetivo:**

Avaliar os efeitos das variações do ANP em resposta à intervenção interdisciplinar para perda de peso na Síndrome Metabólica (SMet) e nos riscos cardiometabólicos em adolescentes com obesidade.

**Métodos:**

73 adolescentes com obesidade participaram de uma terapia interdisciplinar para perda de peso de 20 semanas, incluindo uma abordagem clínica, nutricional, psicológica e de exercícios físicos. A composição corporal, análises bioquímicas e pressão sanguínea foram avaliadas. A SMet foi classificada de acordo com a Federação Internacional de Diabetes (IDF) (2007). Após o tratamento, os voluntários foram divididos de acordo com os níveis de plasma do ANP aumento (n=31) ou ANP redução (n=19).

**Resultados:**

Ambos os grupos apresentaram redução significativa de peso corporal, índice de massa corporal (IMC) e circunferências de cintura, pescoço e quadril (CC, CP e CQ, respectivamente), e aumento da massa livre de gordura (MLG). É interessante observar que houve uma redução significativa na gordura corporal, na razão de TG/HDL-c e na prevalência de SMet (de 23% para 6%) somente no grupo com ANP aumento.

**Conclusão:**

Este estudo sugere que o aumento nos níveis séricos de ANP após a terapia para perda de peso pode estar associado a melhorias nos riscos cardiometabólicos e na prevalência reduzida de SMet em adolescentes com obesidade.

## Introdução

Classificada como um problema de saúde pública, a obesidade é caracterizada pelo acúmulo excessivo do tecido adiposo, em grande parte gerado pelo desequilíbrio energético causado pelo estilo de vida sedentário e o maior consumo de alimentos calóricos, com impacto negativo na saúde física e emocional.^1 , 2^ Nos últimos anos, a prevalência de sobrepeso em crianças e adolescentes aumentou em 60%, tornando-se um fator preocupante para a saúde pública de gerações futuras.^3^

Os peptídeos natriuréticos (NPs): peptídeo natriurético atrial (ANP); peptídeo natriurético ventricular tipo B (BNP); e peptídeo natriurético tipo C (CNP), liberados pelas células vasculares, são hormônios produzidos pelo coração. Suas ações tradicionalmente conhecidas são a natriurese, a diurese e a vasodilatação que, juntas, neutralizam o estresse cardíaco excessivo.^4 - 6^

Embora sejam considerados como hormônios cardiovasculares, duas décadas atrás os receptores dos peptídeos natriuréticos foram encontrados no tecido adiposo de ratos e humanos.^7^ Assim, o ANP esteve relacionado ao aumento da atividade lipolítica em adipócitos humanos e como um indutor do escurecimento ( *browning* ).^6 , 8^ Além disso, estudos demonstraram uma relação inversa entre os níveis circulantes de peptídeos natriuréticos e o índice de massa corporal (IMC), mostrando que o nível circulante de ANP é reduzido em indivíduos com obesidade e que está positivamente associado ao aumento da oxidação de gordura e perda de peso.^5 , 9^

Também foi demonstrado que exercícios físicos agudos e regulares, assim como uma dieta saudável e normal, têm um grande impacto na liberação de ANP em adultos com obesidade.^9 , 10^ Assim, as ações importantes de ANP podem ser restauradas com perda de peso e um estilo de vida saudável por meio de um tratamento interdisciplinar.

Neste contexto, o objetivo deste estudo foi questionar se as mudanças nos níveis de ANP em resposta à terapia clínica interdisciplinar para perda de peso podem melhorar a prevalência da Síndrome Metabólica (SMet) e os riscos cardiometabólicos em adolescentes com obesidade. Nossa hipótese é a de que o aumento das concentrações plasmáticas de ANP, promovidos pela terapia interdisciplinar para perda de peso, pode gerar benefícios metabólicos e impacto na prevalência de SMet em comparação aos voluntários que apresentaram redução de ANP após o tratamento.

## Métodos

### Participantes

Este estudo incluiu 73 Adolescentes com obesidade de ambos os gêneros, com idades entre 14 e 19 anos. O estudo foi anunciado em diferentes mídias: jornais, revistas, rádio e televisão; e o primeiro contato foi feito com os voluntários. A entrevista clínica inicial foi realizada por um endocrinologista para determinar os critérios de inclusão e exclusão. Todos os voluntários apresentaram, como critério de inclusão, estágio de Tanner ≥ V^11^ e IMC > 95% de acordo com Centro de Controle e Prevenção de Doenças.^12^ Os critérios de exclusão foram: doença genética identificada, gravidez, uso prévio de drogas, consumo crônico de álcool, presença de doenças hepáticas virais, outras causas para esteatose hepática, incapacidade de realizar atividades físicas e não ter acesso a meios eletrônicos (telefone celular ou computador). O estudo foi realizado de acordo com os princípios da Declaração de Helsinki, e foi aprovado pelo Comitê de Ética da Universidade Federal de São Paulo (#0052/2016), registrado com o número: RBR-6txv3v.

### Desenho do Estudo

A terapia interdisciplinar consistiu na avaliação clínica, treinamento físico, apoio nutricional e psicológico. Além disso, a intervenção incluiu temas educativos via internet, promovendo mudanças ao estilo de vida e incentivando um comportamento saudável entre os adolescentes.

### Medidas antropométricas e composição corporal

Peso , estatura, IMC e circunferência da cintura, pescoço e quadril (CC, CP e CQ, respectivamente), foram medidos com procedimentos padrão.^13^ Para obter os valores relacionados ao IMC e à taxa metabólica basal, foi realizada análise da composição corporal foi utilizado para avaliar a composição e a massa corporal (massa gorda e massa magra), assim como a taxa metabólica de repouso (TMR), utilizando os princípios da impedância bioelétrica. Após o tratamento, a razão do percentual de gordura corporal (%GC)/ percentual de massa livre de gordura (%MLG) foi calculada.

### Análise sérica

Amostras de sangue foram coletadas após um período de jejum noturno de 12 horas. A amostra foi dividida em soro e plasma, e as concentrações de glicose, insulina, triglicérides (TG), colesterol total (CT), lipoproteína de alta densidade (HDL-c) e lipoproteína de baixa densidade (LDL-c) foram determinadas por métodos enzimático-colorimétricos (CELM, Barueri, Brasil). O ANP foi medido com um kit Elisa da R&D Systems (Minneapolis, MN, EUA).

### Diagnóstico da Síndrome Metabólica

Os diagnósticos da SMet foram analisados de acordo com os critérios da Federação Internacional de Diabetes (IDF):^14^ circunferência da cintura maior que o 90º percentil para idade e sexo, associado a dois ou mais parâmetros alterados: valores de HDL-c ≤50 mg/dL para meninas e ≤40 mg/dL para meninos; concentrações de TG maiores que 150 mg/dL; níveis de glicose no sangue maiores que 100 mg/dL, e pressão sanguínea ≥130/85 mmHg.

### Protocolos do tratamento interdisciplinar

Os voluntários foram incluídos em uma abordagem clínica interdisciplinar, com pesquisadores, durante o protocolo. Peso, estatura e circunferências foram avaliados nos cinco encontros. O perfil do sangue e a composição corporal foram avaliados somente no início e após o tratamento. A cada abordagem clínica, os adolescentes tinham prescrições nutricionais adequadas para idade e sexo idade e sexo, uma sessão com um psicólogo e assistência no programa autoguiado de exercícios.

### Terapia interdisciplinar

#### Intervenção clínica

Os voluntários foram ao endocrinologista antes e depois da terapia, com seus pais, para avaliar suas condições de saúde e clínicas, além da maturação sexual.

#### Apoio nutricional

O consumo diário de calorias foi avaliado com o recordatório alimentar de 24 horas (24 HR), realizado no início e ao final das 20 semanas de intervenção. O consumo de energia foi estabelecido considerando os níveis recomendados pela referência de consumo para pessoas com baixos níveis de atividade física, da mesma idade e sexo, seguindo uma dieta balanceada. O software DIETSMART^®^ foi utilizado para analisar o consumo alimentar e para determinar a redução do consumo calórico entre 300 e 500 kcal/dia. A distribuição de macronutrientes foi gordura (25-35%), carboidratos (45-65%) e proteínas (10-30%).^15^

A cada semana, diferentes temas de saúde foram postados no programa online de perda de peso, incluindo aulas sobre alimentação (exemplos: alimentos de baixa caloria, alimentos diet e light, dietas para perda de peso, boas escolhas alimentares nas férias, fins de semana e festas, embalagens dos alimentos e outros tópicos relacionados). Não foram recomendados medicamentos e nem o uso de antioxidantes.

#### Programa de Exercícios

O exercício físico foi escolhido pelo modelo autoguiado, pelo qual o adolescente selecionava os exercícios de acordo com sua preferência.^16^ A escolha era guiada em termos de abordagem clínica por um profissional da área, que avaliava frequência (três vezes/semana) e duração (mínimo de uma hora). Da mesma forma, as variações da composição corporal e a taxa metabólica basal foram consideradas para a escolha da modalidade a ser praticada, para garantir os benefícios no programa da perda de peso.^17^ O nível de atividade física foi monitorado na avaliação inicial e após 5 semanas, utilizando o Questionário Internacional de Atividade Física – versão curta.^18^

Nos temas de saúde do programa virtual, os voluntários tinham acesso a vídeos sobre a realização correta dos exercícios, incluindo frequência, intensidade e volume, ajudando os adolescentes em suas escolhas.

#### Aconselhamento Psicológico

Os adolescentes participaram de seis sessões de terapia em grupo cujo objetivo era ajudá-los a lidar com suas emoções. Temas diferentes associados à obesidade foram abordados de acordo com a progressão do tratamento: depressão, alterações na imagem corporal, ansiedade e baixa autoestima.^19^

#### Educação para a saúde virtual

O grupo recebeu apoio online voltado à educação para a saúde durante as 20 semanas. O programa de perda de peso online foi utilizado para acessar vídeos educacionais semanais que poderiam ajudar no entendimento da questão da obesidade e no processo de emagrecimento, alimentação saudável e mudanças no estilo de vida, divididos em 20 temas com base no e-book *Saber Emagrecer* .^20^

## Análise Estatística

A análise estatística foi realizada utilizando o software STATISTICA, versão 7.0 para Windows (StartSoft, Tulsa OK. EUA). O nível de significância estatística foi p<0,05. A normalidade dos dados foi verificada com o teste de Kolmogorov-Smirnov. Os dados paramétricos foram demonstrados como média ± desvio padrão (DP), e as variáveis que não tinham distribuição normal foram normalizadas com o Escore Z. O teste t foi realizado por comparação entre as medidas no início e após a terapia para toda a amostra. As comparações entre as medidas no início e após o tratamento foram realizadas utilizando o modelo de ANOVA de medidas repetidas, e o teste post-hoc de Fisher para analisar os efeitos da intervenção e a diferença entre o aumento de ANP nos grupos crescente e decrescente. As comparações entre a prevalência da SMet antes e depois da terapia foram verificadas com o teste de qui-quadrado.

Utilizando o software G^∗^Power^®^ 3.0.10, obtivemos uma amostra de 46 voluntários, considerando a análise estatística realizada com o modelo de ANOVA de medidas repetidas. O tamanho do efeito foi 0,30, e o poder foi de 80%, com base no ANP dividido em dois grupos e dois períodos de avaliação (início e 20 semanas após a intervenção).

## Resultados

O protocolo foi iniciado com um número total de 73 adolescentes com obesidade participando da terapia interdisciplinar para perda de peso. Dos 73 pacientes, 50 completaram o tratamento (participação em 75% das intervenções). Após o tratamento, os voluntários foram divididos de acordo com os níveis plasmáticos de ANP aumentados (n=31) e reduzidos (n=19) em relação a medida basal. As desistências ocorreram por conta de fatores como trabalho, estudos e não-aderência aos meios eletrônicos.

### Efeitos dos níveis plasmáticos reduzidos de ANP após a terapia na composição corporal e parâmetros metabólicos

O grupo ANP redução, após o tratamento, apresentou redução significativa de peso, IMC, razão de %GC/%MLG, circunferências da cintura, pescoço e quadril, e aumento da MLG (kg e %) após o tratamento para perda de peso. No perfil metabólico do sangue, só foi possível observar melhorias significativas no HDL-c. Os níveis de ANP foram significativamente menores neste grupo e, em comparação ao grupo aumento de ANP, os valores de ANP no início foram maiores ( Tabela 1 ).

**Tabela 1:** – Composição corporal e antropométrica por grupo, de acordo com os níveis sanguíneos reduzidos e aumentados de ANP, no início e após a terapia interdisciplinar para adolescentes com obesidade

	Redução ANP (n=19)			Aumento ANP (n=31)		
	Início	Após a terapia	Δ	Início	Após a terapia	Δ
Peso (kg)	110,4±16,7	105,7±17,7*	-4,7±3,7	112,5±14,4	105,5±12,1*	-6,9±6,5
IMC (kg/m^2^)	39,2±4,7	37,1±5,0*	-2,0±1,5	37,8±4,4	35,3±4,3*	-2,4±2,1
Gordura corporal (%)	37,8±4,9	36,6±4,0	-1,1±2,4	38,0±5,6	35,1±5,5*	-2,9±3,0^†^
Gordura corporal (kg)	41,6±7,8	38,4±7,6*	-3,1±3,2	42,1±8,9	37,2±7,6*	-5,8±5,9
Massa livre de gordura (%)	62,1±4,8	63,6±3,9*	1,5±2,1	62,3±5,3	64,8±5,5*	2,4±3,0
Massa livre de gordura (kg)	68,6±12,1	67,3±12,4*	-1,2±2,5	70,0±9,2	68,3±8,8*	-1,6±2,3
Razão %GC/%MLG	0,61±0,12	0,58±0,10*	-0,03±0,05	0,62±0,13	0,55±0,12*	-0,06±0,07
TMR (kcal)	2086,5±367,9	2044,2±371,1	-42,3±75,9	2128,4±282,5	2078,9±266,1	-49,4±72,8
CC (cm)	110,9±11,7	107,1±13,7*	-3,8±4,3	109,4±8,3	103,3±9,2*	-6,0±5,6
CQ (cm)	126,9±9,0	123,3±7,9*	-3,6±3,0	125,6±7,4	120,5±7,6*	-5,1±4,8
CP (cm)	40,1±4,2	39,0±4,5*	-1,1±0,9	40,0±3,6	38,6±3,3*	-1,4±1,6

*p<0,05 em comparação ao início; ^†^p<0,05 em comparação ao grupo redução ANP. Os dados são apresentados como média (DP). Valores de referência: Glicose (60–110 mg/dL), Insulina (<20 U/mL); HOMA-IR (<2,0); Colesterol total (<17 mg/dL); HDL-c (>30 mg/dL); LDL-c (<130 mg/dL); Triglicérides (33–12 mg/dL). IMC: Índice de Massa Corporal; razão %GC/%MLG: razão do percentual de gordura corporal / % massa livre de gordura; TMR: taxa metabólica de repouso; CC: circunferência da cintura; CQ: circunferência do quadril; CP: circunferência do pescoço.

### Efeitos dos níveis plasmáticos aumentados de ANP após a terapia na composição corporal e nos parâmetros metabólicos

O grupo que aumentou o ANP apresentou redução significativa de peso, IMC, razão de %GC/MLG, circunferências de cintura, pescoço e quadril, e MLG crescente (kg e %) após a terapia para perda de peso. A redução da gordura corporal (%) só foi observada neste grupo. Foi possível observar melhorias significativas no HDL-c e, considerando a relação entre HDL-c e triglicérides representada pela razão TG/HDL-c, uma redução significativa foi observada. Os níveis de ANP foram significativamente maiores ao comparar os momentos iniciais e após o tratamento ( Tabela 2 ).

**Tabela 2:** – Parâmetros metabólicos por grupo de acordo com os níveis sanguíneos reduzidos e aumentados de ANP no início e após o tratamento interdisciplinar para adolescentes com obesidade

	redução ANP (n=19)			Aumento ANP (n=31)		
	Início	Após a terapia	Δ	Início	Após a terapia	Δ
PAS	121,3±11,2	122,1±11,3	0,3±12,1	116,7±8,3	117,9±8,2	-0,3±7,1
PAD	78,6±7,7	78,9±9,3	0,8±13,3	75,4±,.7	75,2±5,1	1,2±8,4
Glicose (mg/dL)	88,7±10,3	89,6±7,3	0,9±11,1	90,4±5,9	88,0±7,4	-1,6±8,9
Insulina (U/dL)	19,9±16,9	20,3±18,7	0,4±14,4	18,3±7,0	16,4±7,8	-1,9±7,6
HOMA-IR	4,3±3,8	4,6±4,3	0,2±3,5	4,1±1,6	3,6±1,7	-0,5±1,8
C-total (mg/dL)	159,4±35,2	158,0±41,6	-1,3±27,9	162,2±33,0	155,1±33,0	-7,1±38,4
HDL-c (mg/dL)	43,1±9,8	46,0±9/,5*	2,9±5,9	38,1±7,4	41,3±7,4*	3,1±5,6
LDL-c (mg/dL)	91,6±33,7	89,0±37,8	-2,6±22,7	97,3±27,9	91,1±28,3	-6,2±29,7
TG (mg/dL)	123,4±50,6	103,0±61,4	-20,3±62,5	133,1±66,6	113,3±58,8	-19,7±62,0
Razão TG/HDL-c	3,0±1,6	2,3±1,4	-0,7±1,5	3,7±2,2	2,9±1,7*	-0,7±1,7
ANP (pg/mL)	435,5±250,1	328,7±243,5*	-106,6±136,5	245,2±194,8^†^	394,2±236,7*	149,0±136,2

*p<0,05 em comparação ao início; ^†^p<0,05 em comparação ao redução ANP; PAS: Pressão Arterial Sistólica; PAD: Pressão Arterial Diastólica; TG: Triglicérides; ANP: Peptídeo Natriurético Atrial.

### Comparação entre Grupos

A comparação entre os valores delta por grupos mostrou que somente a gordura corporal Δ foi maior no grupo que aumentou em comparação ao grupo que reduziu o ANP ( Tabela 2 ).

Efeitos dos níveis plasmáticos decrescentes e crescentes de ANP após o tratamento na prevalência da síndrome metabólica

**Figura 1:**
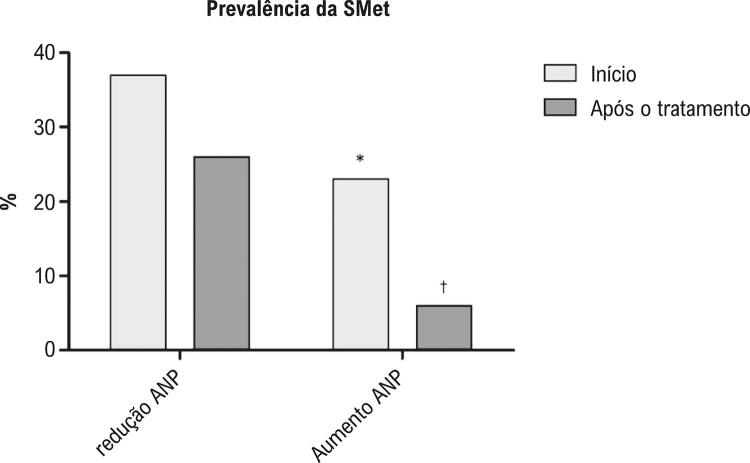
– Prevalência da SMet. *Diferença no início em comparação ao redução ANP; †Diferença entre o início e o pós-terapia no mesmo grupo.

No início, a prevalência da SMet foi maior no grupo redução ANP em comparação ao grupo aumento ANP. Após 20 semanas de terapia interdisciplinar, a prevalência da SMet diminuiu de 37% para 26% (p=0,10) no grupo redução ANP, e de 23% para 6% no grupo aumento ANP (p=0,00) ( Figura 1 ).

## Discussão

O objetivo desta investigação foi avaliar o papel dos níveis plasmáticos reduzidos e aumentados pós terapia ANP e outros riscos cardiometabólicos em adolescentes com obesidade que participaram de um tratamento interdisciplinar para perda de peso. Ambos os grupos apresentaram redução significativa no peso corporal e no IMC, assim como melhorias na MLG e HDL-c, o que mostra a importância desta abordagem clínica para melhorar as condições de saúde de adolescentes com obesidade.

**Figura 2:**
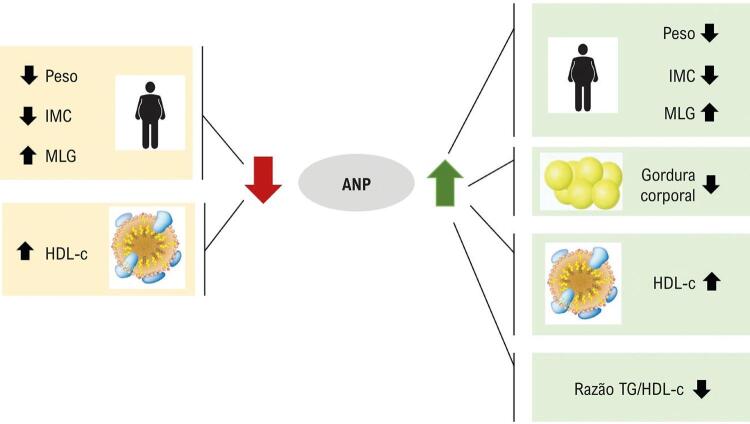
Destaques separados pelos grupos Aumento e redução do ANP.

É interessante observar que somente os adolescentes que apresentaram níveis aumentados de ANP tiveram redução significativa da prevalência da SMet (de 23% para 6%), associada à redução no TG/HDL-c e gordura corporal, após o tratamento para perda de peso (todos os resultados estão detalhados na Figura 2 ).

A SMet pode ser definida como um grupo de alterações, incluindo: hipertensão, dislipidemia, obesidade abdominal e resistência à insulina; e é considerada como uma importante comorbidade da obesidade, com prevalência de aproximadamente 32% em crianças e adolescentes com obesidade.^11 , 21 , 23^ A presença da SMet em faixas etárias mais novas pode ser responsável por aumentar as chances de desenvolver diabetes mellitus tipo II em cinco vezes, e as chances de aumentar a mortalidade geral em 1,6 vezes, incluindo 44% das doenças cardiovasculares.^14^

Santhekadur et al.,^8^ recentemente mostraram como a ocorrência da SMet está associada à presença reduzida de hormônios do coração, como os peptídeos natriuréticos (ANP, BNP e CNP), e à expressão alterada em seus receptores; assim prejudicando suas funções benéficas no cérebro, coração, músculos esqueléticos, tecido adiposo, pâncreas, rins e fígado, contribuindo com a gênese e manutenção da SMet.

Na verdade, a função primária do ANP está relacionada aos efeitos cardiovasculares. Este hormônio circulante de origem cardíaca tem hemodinâmica relevante e ações antirremodeladoras, além de um papel importante na regulação do volume sanguíneo intravascular e do tônus vascular por meio da promoção da natriurese e da diurese no rim. Atua também no relaxamento dos músculos vasculares lisos, regulando, assim, o volume e pressão sanguíneos.^6 , 24^ O papel benéfico dos peptídeos tem uma influência direta na resistência à insulina e menos tolerância à glicose.^25^

Anteriormente, Wang et al.,^26^ descreveram que o nível circulante de ANP é reduzido em indivíduos com obesidade, e que a perda de peso pode promover um aumento neste peptídeo natriurético. Com base na variabilidade interindividual observada na nossa amostra com relação às respostas de ANP à intervenção, comparamos os adolescentes com níveis de ANP decrescentes e crescentes, independentemente da perda de peso. De acordo com os nossos resultados, a porcentagem de gordura corporal só foi significativamente reduzida no subgrupo que apresentou níveis séricos aumentados de ANP. Este resultado pode estar conectado à função mais recentemente descrita de ANP, que promove um aumento na transcrição do gene da UCP-1, mecanismo relevante associado ao gasto de energia, produção de calor, com um possível papel na termogênese e no escurecimento do tecido adiposo (browning).^5 , 9^

Sabemos que este é o primeiro estudo a avaliar os efeitos dos níveis séricos crescentes e decrescentes de ANP na composição corporal e no risco metabólico, de acordo com a terapia interdisciplinar para perda de peso em adolescentes com obesidade. Juntos, os resultados demonstrados no grupo ANP crescente podem parcialmente explicar a redução significativa da SMet somente neste grupo (de 23% para 6%); assim contribuindo com os mecanismos básicos que conectam a obesidade e a saúde cardiometabólica nos primeiros estágios do desenvolvimento. Para corroborar esta informação, Masquio et al.,^27^ recentemente demonstraram que a prevalência de SMet em adolescentes com obesidade pode impactar a redução da espessura da camada íntima-média de artérias carótidas após a terapia para perda de peso. Por fim, esses resultados podem ser importantes para a prática clínica, considerando esta população analisada.

Além disso, a presença da SMet está fortemente correlacionada ao status nutricional,^28 , 29^ enfatizando a importância da redução significativa no IMC e na massa de gordura corporal; e um aumento na MLG em um contexto de obesidade. IMC e CC são medidas importantes para determinar a prevalência de SMet; mas o percentual e distribuição de gordura podem ser indicadores melhores para a avaliação clínica. A obesidade abdominal é o componente mais frequentemente observado da SMet, e o acúmulo central de gordura corporal está associado à resistência à insulina, enquanto a distribuição de gordura corporal periférica tem impacto metabólico relativamente menor.^30 , 31^

As concentrações de HDL-c, além de fazerem parte do diagnóstico da SMet, são um preditor independente e inverso para doenças cardiovasculares. As funções do HDL-c são relacionadas à proteção em potencial contra a doença arterial, sendo mais conhecidas por sua habilidade de promover o efluxo de colesterol da parede arterial. As partículas de HDL-c têm propriedades que reduzem a oxidação, a inflamação vascular e a trombose, além de melhorar a função endotelial, promover o reparo endotelial, melhorar a sensibilidade à insulina e promover a secreção de insulina pelas células-beta pancreáticas.^32 - 34^

Embora os níveis de HDL-c estejam altos em ambos os grupos, uma redução significativa na razão de TG/HDL-c foi observada no grupo ANP crescente, somente. A razão de TG/HDL-c é uma medida única que integra a informação sobre resistência à insulina e medidas aterogênicas de lipídios com relação ao risco cardiovascular, podendo prever o desenvolvimento de doença coronariana e mortalidade cardiovascular.^35 , 36^

Este estudo aparenta ser o primeiro a comparar o efeito de do aumento e da redução dos níveis de ANP em reposta à intervenção para perda de peso em adolescentes com obesidade. Nossos resultados são preliminares, e sua interpretação deve ser analisada com cuidado. Principalmente porque a amostra reduzida requer outros estudos incluindo mais participantes. Da mesma forma, teria sido importante avaliar um grupo controle não obeso, sem intervenção.

## Conclusão

De acordo com este estudo preliminar, adolescentes com obesidade e com aumento das concentrações de ANP em resposta à intervenção interdisciplinar apresentam evidências de redução da prevalência da SMet, o que pode contribuir para melhorar a saúde cardiometabólica nesta população.
