# Supplementary material for: O Papel dos Níveis Séricos de ANP na Perda de Peso, Risco Cardiometabólico e Composição Corporal de Adolescentes com Obesidade Submetidos a Terapia Interdisciplinar
Source: Arq Bras Cardiol. 2021 Nov 17;118(1):33–40. [Article in Portuguese] doi: 10.36660/abc.20200735 (PMC8959045; doi:10.36660/abc.20200735)
Supplement: Translation

# Translation: The Role of ANP Level Response in Interdisciplinary Weight Loss Therapy on Cardiometabolic Risk and Body Composition in Adolescents with Obesity

**Authors:** Ana Claudia Pelissari Kravchychyn, Raquel Munhoz da Silveira Campos, Yasmin Alaby Martins Ferreira, Sofia Emanuelle de Castro Ferreira Vicente, Flávia Campos Corgosinho, Lila Missae Oyama, Valter Tadeu Boldarine, Lian Tock, David Thivel, Ana Raimunda Dâmaso

**Affiliations:** 1 Brazil Universidade Federal de São Paulo - Programa de Pós-graduação em Nutrição, São Paulo, SP – Brazil; 2 Brazil Universidade Federal de São Paulo - Departamento de Biociências Campus Baixada Santista - Programa de Pós Graduação Interdisciplinar em Ciências da Saúde, Santos, SP - Brazil; 3 Brazil Anhanguera Educacional Ltda, Guarulhos, SP – Brazil; 4 Brazil Universidade Federal de Goiás – Nutrição, Goiânia, GO – Brazil; 5 France Université Clermont Auvergne, Clermont-Ferrand, Auvergne-Rhône-Alpes – France

**Keywords:** Metabolic Syndrome, Atrial Natriuretic Peptide, Obesity, Adolescent, Weight Loss, Insulin Resistance, Metabolism

## Abstract

**Background:**

The action of atrial natriuretic peptide (ANP) on natriuresis, diuresis and vasodilatation, insulin resistance, liver, kidney, and adipose tissue may contribute to the healthy metabolic and cardiovascular development. Even though the circulating level of ANP is reduced in patients with obesity, its response to weight loss remains poorly explored in pediatric populations.

**Objective:**

To evaluate the effects of ANP variations in response to interdisciplinary weight loss intervention on metabolic syndrome (MetS) and cardiometabolic risks in adolescents with obesity.

**Methods:**

73 adolescents with obesity attended a 20-week clinical interdisciplinary weight loss therapy including clinical, nutritional, psychological and exercise training approach. Body composition, biochemical analyses and blood pressure were evaluated. MetS was classified according to the International Diabetes Federation (IDF) (2007). After the treatment, volunteers were divided according to Increasing (n=31) or Decreasing (n=19) ANP plasma levels.

**Results:**

Both groups present significant reduction of body weight, Body Mass Index (BMI), waist, neck and hip circumferences (WC, NC and HC, respectively) and increasing fat-free mass (FFM). Interestingly, a significant reduction in body fat, TG/HDL-c ratio and MetS prevalence (from 23% to 6%) was observed in the Increased ANP group only.

**Conclusion:**

This study suggests that an increase in ANP serum levels after weight loss therapy could be associated with improvements in cardiometabolic risks and the reduced prevalence of MetS in adolescents with obesity.

## Introduction

Classified as a public health disease, obesity is characterized by the excessive accumulation of adipose tissue, mostly generated by energy imbalance due to sedentary lifestyles and increased consumption of high-calorie foods, with a negative impact on physical and emotional health.^1 , 2^ In the past years, the prevalence of overweight in children and adolescents has increased by 60%, leading to worrying public health issues for future generations.^3^

The natriuretic peptides (NP): Atrial Natriuretic Peptide (ANP); Ventricular Natriuretic Peptide, B-type (BNP); and natriuretic peptide, C-type (CNP), released by vascular cells, are hormones produced by the heart. Their traditionally known actions are natriuresis, diuresis and vasodilatation, which, together, neutralize excessive heart stress.^4 - 6^

Although classically considered as cardiovascular hormones, two decades ago NP receptors were found in the adipose tissue of both rats and humans.^7^ Therefore, ANP was related to the increasing lipolytic activity in human adipocytes and as a browning inducer.^6 , 8^ In addition, studies showed an inverse relationship between circulating NP levels and Body Mass Index (BMI), showing that the circulating level of ANP is reduced in obese individuals and positively associated with the increase in fat oxidation and weight loss.^5 , 9^

It has been shown that acute and regular physical exercise, as well as a healthy, normal diet, have a huge impact on ANP release in adults with obesity.^9 , 10^ Thus, the important actions of ANP can be restored with weight loss and a healthy lifestyle through an interdisciplinary treatment.

In that context, the aim of the present study was to question whether or not ANP level changes in response to clinical interdisciplinary weight loss therapy may contribute to improve the metabolic syndrome (MetS) prevalence and cardiometabolic risks in adolescents with obesity. We hypothesize that increasing plasma levels of ANP promoted by interdisciplinary therapy for weight loss have greater beneficial effects on cardiometabolic risks and impact the prevalence of MetS in comparison to volunteers who presented decreasing ANP after treatment.

## Methods

### Participants

This study included 73 obese adolescents of both genders aged from 14 to 19 years. The study was announced in the different media: journals, magazines, radio, and television; and the first contact with volunteers was made. The initial clinical interview was performed by an endocrinologist to determine the inclusion and exclusion criteria. All volunteers presented the inclusion criteria of post-pubertal Tanner Stage ≥ V^11^ and BMI > 95% in the Center for Diseases Control.^12^ The exclusion criteria were: identified genetic disease, pregnancy, previous drug use, chronic alcohol use, presence of viral hepatic diseases, other causes of liver steatosis, inability to perform physical activities and no access to any electronic means (cell phone or computer). The study was conducted according to the principles of the Declaration of Helsinki, and it was approved by the ethics committee *Universidade Federal de São Paulo* (#0052/2016), being registered with Brazilian Clinical Trial registration number: RBR-6txv3v.

### Research design

The classical interdisciplinary therapy consisted of clinical assessment, exercise training, nutritional and psychological support. Additionally, the intervention included web-based education themes promoting lifestyle changes to encourage a healthy behavior among adolescents.

### Anthropometric measurements and body composition

Weight, height, BMI, and waist, neck and hip circumference (WC, NC and HC, respectively), were measured using standard procedures.^13^ In order to obtain the values related to BMI and the basal metabolic rate, a body composition analyzer was used to assess body mass and composition (fat and lean body mass), as well as the resting metabolic rate (RMR), using bioelectrical impedance principles. Post treatment, percent body fat (%BF)/ percent fat-free mass (%FFM) ratio was calculated.

### Serum analysis

Blood samples were collected after an overnight fasting period (12 hours). Serum was separated into serum and plasma, and concentrations of glucose, insulin, triglycerides (TG), total cholesterol (TC), high density lipoprotein-cholesterol (HDL-c), low density lipoprotein-cholesterol (LDL-c) were determined by enzymatic colorimetric methods (CELM, Barueri, Brazil). ANP was measured with an enzyme-linked immunosorbent assay (ELISA) kit from R&D Systems (Minneapolis, MN, USA).

### Metabolic syndrome diagnosis

The MetS diagnoses were analyzed according to International Diabetes Federation (IDF) criteria:^14^ waist circumference being higher than the 90^th^percentile for age and gender, and associated with two or more altered parameters: HDL-c values ≤50 mg/dL for girls and ≤40 mg/dL for boys; concentrations of TG higher than 150 mg/dL; blood glucose levels higher than 100 mg/dL, and blood pressure ≥130/85 mmHg.

### Interdisciplinary treatment protocols

Volunteers were involved in an interdisciplinary clinical approach with researchers throughout the protocol. Weight, height, and circumferences were evaluated in the five meetings. Serum blood profile and body composition were evaluated only at the baseline and after treatment. At each clinical approach, the adolescents had low-calorie dietary prescriptions per age and gender, a session with a psychologist and assistance in the self-guided exercise program.

### Interdisciplinary therapy

#### Clinical intervention

The volunteers visited the endocrinologist before and after therapy with their parents to address their health, clinical conditions, and sexual maturation.

#### Nutritional Support

Daily intake of calories was assessed using a self-reported 24-hour dietary recall (24 HR) completed at baseline and at the end of the 20-week intervention. Energy intake was set at levels recommended by the dietary reference intake for subjects with low levels of physical activity and of the same age and gender following a balanced diet. The DIETSMART^®^software was used to analyze dietary intake and to determine the reducing caloric intake between 300 and 500 kcal/day. The distribution of macronutrients was fat (25-35%), carbohydrate (45-65%) and protein (10-30%).^15^

Every week, different health themes were posted in the online weight loss program with instruction dietary lessons (Example: low-calorie foods, diet and light foods, weight loss diets, good food choices on holidays, weekends and celebrations, food labels and other related topics). No pharmacotherapies or antioxidant were recommended.

#### Exercise program

Physical exercise was chosen by the self-guided method, in which the adolescent selected exercises according to personal pleasure.^16^ The choice was guided, in terms of clinical approach, by a professional in this field for frequency (tree times/week) and duration (minimum of one hour/session), and variables of body composition and basal metabolic rate were considered for choosing the modality to be practiced, in order to guarantee benefits for the weight loss program.^17^ The physical activity level was monitored at the initial evaluation and after every 5 weeks using the International Physical Activity Questionnaire (IPAQ - short version).^18^

In the online program health themes, volunteers had access to videos about the correct performance of physical exercises, including frequency, intensity and volume, to help them in their choices.

#### Psychological Counseling

The adolescents attended six therapy group sessions that aimed at helping them deal with their emotions. Different themes associated with obesity were approached according to the progression of the treatment: depression, disturbances of body image, anxiety and decreased self-esteem.^19^

#### Web-based health education

The group received web-based support to health education during the 20 weeks. The online weight loss program was used to access weekly educational videos that would help to understand the obesity issue and the slimming process, healthy eating and changes in lifestyle divided into 20 themes based on the e-book *Saber Emagrecer.* ^20^

## Statistical analysis

Statistical analysis was performed using the software STATISTICA, version 7.0 for Windows (StartSoft, Tulsa OK. USA). The level of statistical significance was set at p<0.05. Data normality was verified using the Kolmogorov-Smirnov test. Parametric data were expressed as mean ± standard deviation (SD), and variables that did not have a normal distribution were normalized by the Z-score. The t-test was performed by comparison between measures at the beginning and after therapy for the entire sample. Comparisons between measures at the baseline and afterwards were made using the repeated measures ANOVA (two-way ANOVA) and the Fisher’s post-hoc test to analyze the effects of intervention and difference between the ANP Increase and Decrease group. Comparisons between the prevalence of MetS before and after therapy was verified by the chi-square test.

Using the G^∗^Power^®^3.0.10 software, we obtained a sample of 46 volunteers, considering the statistical analysis carried out with the repeated measures ANOVA (two-way ANOVA). The effect size was 0.30, and power was 80%, based on ANP divided in two groups and two periods of evaluation (baseline and 20 weeks after the intervention).

## Results

We started with a total number of 73 obese adolescents engaged in interdisciplinary therapy for weight loss. Out of the 73 patients, 50 completed the weight loss therapy (attendance in 75% of treatment interventions). After treatment, the volunteers were divided according to Increasing (n=31) or Decreasing (n=19) ANP plasma levels. The dropouts occurred due to factors such as work, studies, and non-adherence to electronic means. It important to note that no differences were observed in any variables between the completers and non-completers.

### Effects of decreasing ANP plasma levels after therapy on body composition and metabolic parameters

The *decreased ANP group,* post treatment, presented significant reduction of weight, BMI, %BF/%FFM ratio, waist, neck, and hip circumferences, and increasing FFM (kg and %) after weight loss therapy. In the metabolic blood profile, it was only possible to observe significant improvements in HDL-c. ANP levels were significantly lower in this group and, when compared with the *Increasing ANP group,* the values of ANP at baseline were higher (Table 1 ).

**Table 1:** – Anthropometric and Body composition per group according to Decreasing and Increasing ANP blood levels at baseline and after interdisciplinary therapy in adolescents with obesity

	Decreasing ANP (n=19)			Increasing ANP (n=31)		
	Baseline	After therapy	Δ	Baseline	After therapy	Δ
Weight (kg)	110.4±16.7	105.7±17.7*	-4.7±3.7	112.5±14.4	105.5±12.1*	-6.9±6.5
BMI (kg/m^2^)	39.2±4.7	37.1±5.0*	-2.0±1.5	37.8±4.4	35.3±4.3*	-2.4±2.1
Body fat (%)	37.8±4.9	36.6±4.0	-1.1±2.4	38.0±5.6	35.1±5.5*	-2.9±3.0^†^
Body fat (kg)	41.6±7.8	38.4±7.6*	-3.1±3.2	42.1±8.9	37.2±7.6*	-5.8±5.9
Free fat mass (%)	62.1±4.8	63.6±3.9*	1.5±2.1	62.3±5.3	64.8±5.5*	2.4±3.0
Free fat mass (kg)	68.6±12.1	67.3±12.4*	-1.2±2.5	70.0±9.2	68.3±8.8*	-1.6±2.3
%BF/%FFM ratio	0.61±0.12	0.58±0.10*	-0.03±0.05	0.62±0.13	0.55±0.12*	-0.06±0.07
RMR (kcal)	2086.5±367.9	2044.2±371.1	-42.3±75.9	2128.4±282.5	2078.9±266.1	-49.4±72.8
WC (cm)	110.9±11.7	107.1±13.7*	-3.8±4.3	109.4±8.3	103.3±9.2*	-6.0±5.6
HC (cm)	126.9±9.0	123.3±7.9*	-3.6±3.0	125.6±7.4	120.5±7.6*	-5.1±4.8
NC (cm)	40.1±4.2	39.0±4.5*	-1.1±0.9	40.0±3.6	38.6±3.3*	-1.4±1.6

*p<0.05 compared to Baseline;^†^p<0.05 compared to the Decreasing ANP group. Data are presented as mean (SD). Reference values: Glucose (60–110 mg/dL), Insulin (<20 U/mL); HOMA-IR (<2.0); Total cholesterol (<17 mg/dL); HDL-c (>30 mg/dL); LDL-c (<130 mg/dL); Triglycerides (33–12 mg/dL). BMI: Body Mass Index; %BF/%FFM ratio: percent body fat / % fat-free mass ratio; RMR: resting metabolic rate; WC: waist circumference; HC: hip circumference; NC: neck circumference.

### Effects of increasing ANP plasma levels after therapy on body composition and metabolic parameters

The *Increasing ANP group* presented significant reduction of weight, BMI, %BF/%FFM ratio, waist, neck, and hip circumferences, and increasing FFM (kg and %) after weight loss therapy. Body fat (%) reduction was only observed in this group. It was possible to see significant improvements in HDL-c and, considering the relationship between HDL-c and Triglycerides represented by the TG/HDL-c ratio, a significant reduction was observed. ANP levels were significantly higher when comparing the moments of baseline and after therapy ( Table 2 ).

**Table 2:** – Metabolic parameters by groups according to Decreasing and Increasing ANP blood levels at baseline and after interdisciplinary therapy in adolescents with obesity

	Decreasing ANP (n=19)			Increasing ANP (n=31)		
	Baseline	After therapy	Δ	Baseline	After therapy	Δ
SBP	121.3±11,2	122.1±11.3	0.3±12.1	116.7±8.3	117.9±8.2	-0.3±7.1
DBP	78.6±7.7	78.9±9.3	0.8±13.3	75.4±5.7	75.2±5.1	1.2±8.4
Glucose (mg/dL)	88.7±10.3	89.6±7.3	0.9±11.1	90.4±5.9	88.0±7.4	-1.6±8.9
Insulin (U/dL)	19.9±16.9	20.3±18.7	0.4±14.4	18.3±7.0	16.4±7.8	-1.9±7.6
HOMA-IR	4.3±3.8	4.6±4.3	0.2±3.5	4.1±1.6	3.6±1.7	-0.5±1.8
Total-c (mg/dL)	159.4±35.2	158.0±41.6	-1.3±27.9	162.2±33.0	155.1±33.0	-7.1±38.4
HDL-c (mg/dL)	43.1±9.8	46.0±9/.5*	2.9±5.9	38.1±7.4	41.3±7.4*	3.1±5.6
LDL-c (mg/dL)	91.6±33.7	89.0±37.8	-2.6±22.7	97.3±27.9	91.1±28.3	-6.2±29.7
TG (mg/dL)	123.4±50.6	103.0±61.4	-20.3±62.5	133.1±66.6	113.3±58.8	-19.7±62.0
TG/HDL-c ratio	3.0±1.6	2.3±1.4	-0.7±1.5	3.7±2.2	2.9±1.7*	-0.7±1.7
ANP (pg/mL)	435.5±250.1	328.7±243.5*	-106.6±136.5	245.2±194.8^†^	394.2±236.7*	149.0±136.2

*p<0.05 compared to Baseline;^†^p<0.05 compared to Decreasing ANP; SBP: systolic blood pressure; DBP: diastolic blood pressure; TG: Triglycerides; ANP: Atrial Natriuretic Peptide.

### Comparison between Groups

The comparison between delta values by groups showed that only Δ body fat was higher in the *Increasing ANP* group compared to the *Decreasing ANP* group ( Table 2 ).

### Effects of decreasing and increasing ANP plasma levels after therapy on the prevalence of metabolic syndrome

At baseline, the MetS prevalence was higher in the decreasing ANP group compared to the increasing ANP group. After 20 weeks of interdisciplinary therapy, the prevalence of MetS decreased from 37% to 26% (p=0.10) in the decreasing ANP group, and from 23% to 6% in the increasing ANP group (p=0.00) ( Figure 1 ).

**Figure 1:** – Prevalence of MetS. *Difference at baseline compared to Decreasing ANP;†Difference between baseline and after in same group.

## Discussion

The aim of the present investigation was to evaluate the role of *increasing and decreasing ANP plasma levels* on MetS and other cardiometabolic risks in adolescents with obesity who attended interdisciplinary weight loss therapy. Both groups present significant reduction in body weight and BMI and improvements in FFM and HDL-c, showing the importance of this clinical approach in improving the health conditions of adolescents with obesity.

Interestingly, only the adolescents who presented with *increased ANP* levels showed a significant reduction of the MetS prevalence (from 23% to 6%), associated with a decrease in TG/HDL-c and body fat, after the weight loss treatment (all results are detailed in Figure 2 ).

**Figure 2:** The highlights separated by the decreasing and the increasing ANP groups.

MetS can be defined as a cluster of alterations, including: hypertension, dyslipidemia, abdominal obesity and insulin resistance; and is considered as an important comorbidity of obesity, with prevalence of about 32% among children and adolescents with obesity.^11 , 21 , 23^ The presence of MetS at an early age may be responsible for increasing the chances of developing diabetes mellitus type II in five times, and the chances to increase general mortality in 1.6 times, including 44% of cardiovascular diseases.^14^

Santhekadur et al.^8^ recently showed how the occurrence of MetS is associated with the reduced presence of cardiac hormones, such as natriuretic peptides (ANP, BNP and CNP) and altered expression in their receptors; thus damaging their beneficial functions in the brain, heart, skeletal muscle, adipose tissue, pancreas, kidney and liver, and contributing with the genesis and maintenance of the MetS.

In fact, the primary function of ANP is related to cardiovascular effects. This circulating hormone of cardiac origin has relevant hemodynamic and anti-remodeling actions, and plays an important role in the regulation of intravascular blood volume and vascular tone through the promotion of natriuresis and diuresis in the kidney and the relaxation of vascular smooth muscles, thereby regulating blood volume and pressure.^6 , 24^ The beneficial role of cardiac peptides in diabetes has been described, and any decrease in these peptides has a direct influence on insulin resistance and decreased glucose tolerance.^25^

Previously, Wang et al.^26^ described that the circulating level of ANP is reduced in obese individuals, and that weight loss can promote an increase of this natriuretic peptide. Based on the inter-individual variability observed in our sample regarding ANP responses to the intervention, we compared the adolescents who had *decreased* versus *increased ANP levels,* independently of weight loss. According to our results, the percentage of body fat was only significantly reduced in the subgroup that presented *increased* ANP serum levels. This result may be linked to the most recently described function of ANP, which promotes an increase in UCP-1 gene transcription, a relevant mechanism associated with energy expenditure, heat production, and with a possible role in the thermogenesis and adipose tissue browning.^5 , 9^

To our knowledge, this is the first study to evaluate the effects of *increasing and decreasing* serum ANP levels on body composition and cardiometabolic risk following interdisciplinary weight loss therapy in adolescents with obesity. Together, the results shown in the *increasing ANP group* can partially explain the significant reduction in MetS prevalence only in this group (from 23% to 6%); thus contributing with the underlying mechanisms connecting obesity and cardiometabolic health in the early stage of life development. To corroborate this information, Masquio et al.^27^ recently showed that the presence of MetS in adolescents with obesity may impair the reduction in the carotid intima-media thickness after weight loss therapy. Altogether, these results may be important for the clinical practice considering this analyzed population.

In addition, the presence of MetS is strongly correlated with nutritional status,^28 , 29^ highlighting the importance of a significant reduction in BMI and body fat mass; and an increase in the FFM in a context of obesity. BMI and WC are important measurements to determine the MetS prevalence; but fat percentage and distribution may be better indicators for the clinical evaluation. Abdominal obesity is the most frequently observed component of MetS, and central accumulation of body fat is associated with insulin resistance, whereas the distribution of peripheral body fat has a relatively lower metabolic impact.^30 , 31^

The HDL-c concentrations, besides being part of the MetS diagnosis, is an independent inverse predictor for cardiovascular disease. The functions of HDL-c are related to potential protection against arterial disease, being best known by its ability to promote cholesterol efflux from the artery wall. HDL-c particles have properties that reduce oxidation, vascular inflammation and thrombosis, improve endothelial function, promote endothelial repair, enhance insulin sensitivity, and promote insulin secretion by pancreatic beta islet cells.^32 - 34^

Even though HDL-c levels were high in both groups, a significant decrease in the TG/HDL-c ratio has been observed in the *increasing ANP group* only. The TG/HDL-c ratio may serve as a single measure that integrates information on insulin resistance and atherogenic lipid measurements related to cardiovascular risk, being able to predict the development of coronary heart disease and cardiovascular mortality.^35 , 36^

The present study appears to be the first to compare the effect of *increased vs decreased ANP* in response to weight loss intervention in adolescents with obesity. Our results remain preliminary and their interpretation should be analyzed carefully. Mainly, the reduced sample size calls for further studies including more participants. Similarly, it would have been important to have a non-intervention lean control group.

## Conclusion

According to the present preliminary work, adolescents with obesity with *increased ANP* concentration in response to an interdisciplinary intervention present evidence to reduce the MetS prevalence, which may contribute with improved cardiometabolic health in this population.
